# Electrostimulation improves plant growth and modulates the flavonoid profile in aeroponic culture of *Scutellaria baicalensis* Georgi

**DOI:** 10.3389/fpls.2023.1142624

**Published:** 2023-03-01

**Authors:** Kajetan Grzelka, Adam Matkowski, Sylwester Ślusarczyk

**Affiliations:** ^1^ Department of Pharmaceutical Biology and Biotechnology, Division of Pharmaceutical Biology and Botany, Wroclaw Medical University, Wroclaw, Poland; ^2^ Botanical Garden of Medicinal Plants of the Wroclaw Medical University, Wroclaw, Poland

**Keywords:** electric impulse, baicalin, baicalein, soilless cultivation, flavone glucuronides, electroporation, LCMS (liquid chromatography-mass spectrometry)

## Abstract

*Scutellaria baicalensis* Georgi is a valuable medicinal plant of the *Lamiaceae* family. The roots, *Scutellariae baicalensis radix*, are valued in the traditional medicine of East Asia and are also listed in several pharmacopeias, such as the Chinese and European versions. The roots contain a high amount of flavones, such as baicalein, wogonin and their glucuronides, baicalin and wogonoside, respectively, with rare structures of unsubstituted B-ring. These major constituents are responsible for its pharmacological activity, mainly anti-inflammatory, antiviral, and antitumor, as well as BDZ-receptor modulating. There is a fast-growing demand for both the crude drug and the individual flavonoids obtained from it.

However, the variability of content and composition of flavonoids in the roots is significant and affects pharmaceutical use, and little is known about the influence of various factors on root quality. In our experiments, we use aeroponics to determine the effect of electroporation as an abiotic stressor on plant growth, development, and root mass, as well as on its metabolic profile.

**Results:**

Electroporation significantly impacted plant growth and the content of flavonoids, especially baicalein and wogonin, depending on the treatment parameters. Concentrations of aglycones were increased in at least half of the treatment conditions. The greatest amounts (a 2.5-fold increase compared to controls) were recorded after applying an electrical field characterized by the following parameters: E = 3 kV/cm, t = 100 μs, and N = 10. In conclusion, electrostimulation is an innovative and efficient way to increase plant growth and yield in an aeroponic system, as well as modulate the profile and content of bioactive flavones in the roots. However, the fine-tuning of these parameters, such as the electrical field strength (E), length (t), and number (N) of impulses delivered, is of great importance. It was also shown that cultivation of the experimental plants in aeroponics had a positive impact on their survival and development while being a sustainable and efficient horticultural practice.

## Introduction

Cultivating medicinal plants using soilless systems, such as hydroponics, aims to achieve a higher quality of plant biomass and increased concentrations of bioactive metabolites (Jousse et al., 2010). Moreover, these methods reduce water use, waste production, and general environmental burden. Moreover, they can be economically viable due to material savings and higher yields (Goddek et al., 2019). In our experiment, plants were cultivated in an aeroponic system, which is defined by the International Society for Soilless Culture as “a system where roots are continuously or discontinuously exposed to an environment saturated with fine drops (a mist) of nutrient solution”. This technology has many advantages over conventional hydroponics, such as increased nutrient uptake and improved respiration of roots which have been shown to stimulate metabolic processes. It also intensifies photosynthesis and further decreases water and nutritional medium usage (Goddek et al., 2019; Komosa et al., 2020). Various studies have reported an increase in the product yield of plants grown in aeroponics paired with either similar or higher phenolic content when compared to crops grown in soil (Chandra et al., 2014; Wang et al., 2019; Eldridge et al., 2020). This technology is also regarded as the most efficient and convenient among available methods of plant cultivation (Lakhiar et al., 2018).

In order to further promote the accumulation of phytochemicals, we decided to combine this technique with electrostimulation. In the present study, plants were subjected to pulsed electric fields (PEF), resulting in their electroporation (EP). It is a process of cell permeabilization, i.e., pore creation in the cell plasma membrane that occurs after being challenged by electrical impulses. These impulses are generated by an electroporator and then transferred to the treatment chamber where the studied material is situated. In medical biotechnologies, EP is a commonly used technique for intracellular cargo delivery or irreversible cell membrane disruption. Recently, EP performance has been improved by electrodes and device miniaturization (Choi et al., 2022). When it comes to plant-related applications, this technique is most widely utilized in the extraction process, where PEF treatment improves diffusion and mass transfer, thereby greatly increasing the efficacy of this process and decreasing solvent usage (Poojary et al., 2020). This is a much-desired effect since reducing solvent usage on a commercial scale can have a great positive impact on the environment. It was shown that EP magnified conventional solid-liquid extraction of polyphenols from olive leaves in an eco-friendly way using green solvents (Pappas et al., 2021). Another advantage of this non-thermal treatment technique is that it does not affect the quality of the acquired extracts and at the same time enhances the extraction of both hydro- and lipophilic compounds, as has been demonstrated for tomato peel (Luengo et al., 2014). EP is also used in the drying of plant material, where it has been shown to have a protective effect on plant tissue structure and preservation (Thamkaew et al., 2022). It can also accelerate the drying process and increase the rehydration capacity of the treated material (Kwao et al., 2016). However, our study used a new and original approach to plant electroporation as a means to increase the yield of valuable phytochemicals in plant roots. Previous studies have shown that after carefully adjusting the parameters, EP can stimulate plant growth. Such an effect likely occurs due to a stress response after exposure to PEF which, should the process intensity be too great, may also result in necrosis (Eing et al., 2009). In *Arabidopsis thaliana* (L.) Heynh. treated with varying electroporation parameters, only the application of nanosecond PEF of a 5 kV/cm electric field strength resulted in increased plant growth for all pulse durations and had no lethal effect on the seedlings, unlike under higher intensity treatments (Eing et al., 2009). There was also no significant difference in leaf lettuce development when applying liquid fertilizer before or after treatment, which supports the hypothesis of the direct influence of electroporation. The same study has also proven that the permeabilization of solely plant roots was sufficient to achieve a growth-promoting effect (Sonoda et al., 2014). PEF could also serve as an abiotic stressor used to increase the concentration of phytochemicals, as was highlighted in a study where electroporation of *Taxus chinensis* (Pilg.) Rehder cells resulted in a 30% increase in taxuyunnanine C content (Ye et al., 2004). In ginseng, PEF exposure lead to microstructural changes in root cells, increased ion leaching, and a higher content of total phenols (Kim et al., 2019).

Combining soilless cultivation with electroporation is a poorly researched yet very promising approach which could result in great increases in plant biomass and rate of growth (Sonoda et al., 2014). PEF could contribute to the development of cost-effective, more efficient, and eco-friendly technologies in agri-food and pharmaceutical industries. Also, further development of this approach is envisaged by outlying and optimizing cultivation in soilless cultures paired with EP. Due to the scarcity of literature on this subject matter, combining the aforementioned techniques in the present study is quite innovative and promising.

**Figure 1:**
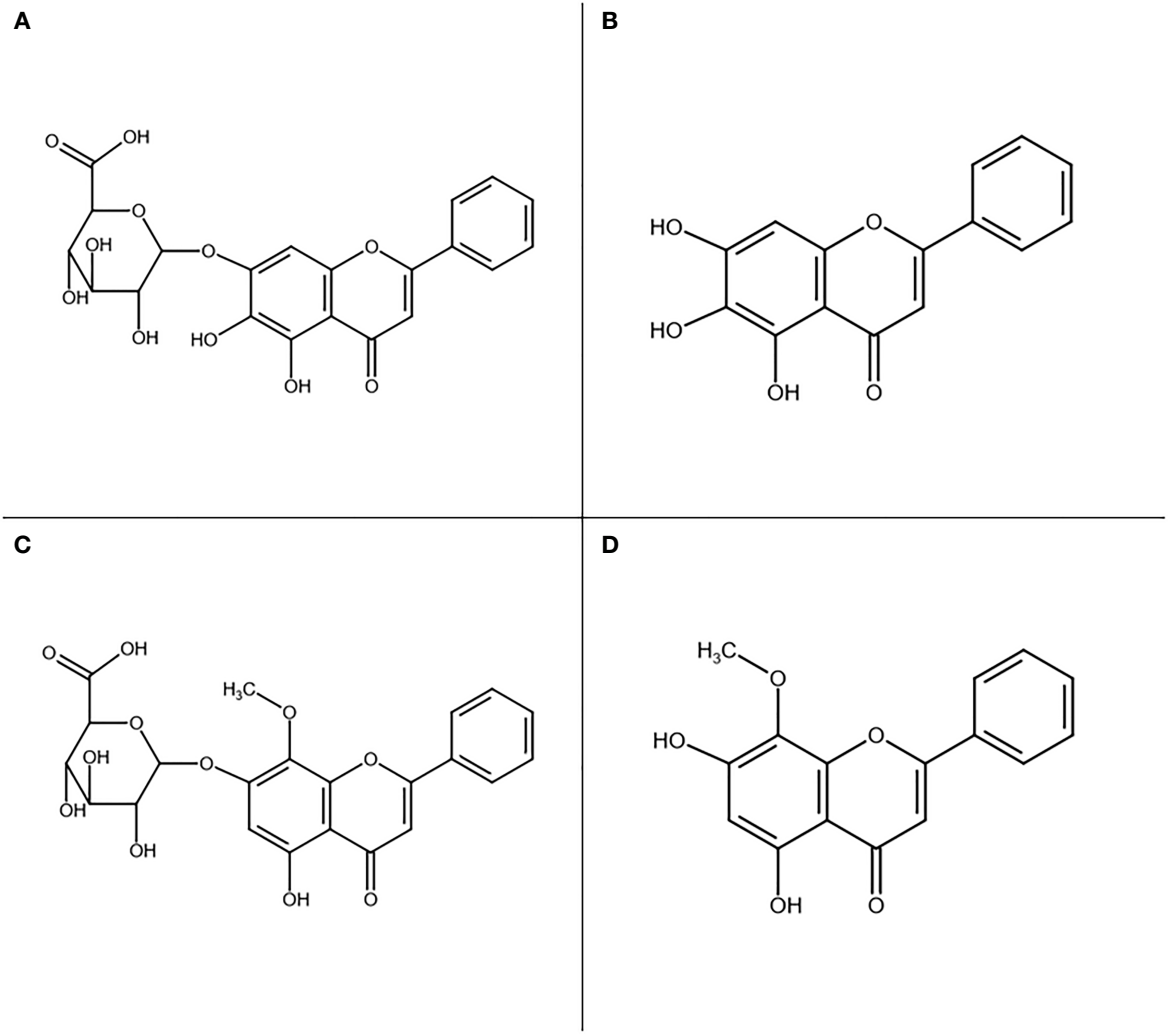
Main active compounds found in Sb root: baicalin **(A)**, baicalein **(B)**, wogonoside **(C)**, and wogonin **(D)**.

*Scutellaria baicalensis* Georgi (abbreviated further in this paper as Sb), a well-known medicinal plant used as a model in our study, is a perennial herb from the *Lamiaceae* family used for centuries in Traditional Chinese Medicine under the name Huang-Qin for treatment of various ailments such as hypertension, liver diseases, inflammation, and respiratory infections (Zhao et al., 2016). This herb has been used increasingly worldwide and is listed in both the Chinese and European Pharmacopoeias as *Scutellariae baicalensis radix*. It has already been developed into several drugs, such as Huangqingan tablets used in hepatitis treatment in China (Wang et al., 2018) and a dental gel used in the treatment of parodontopathy, stomatitis, and oral infections in Poland (Gościniak et al., 2021). The roots are rich in flavonoids that exhibit a broad range of therapeutic effects. Among over 40 such compounds, there are four major active phytochemicals: baicalin, baicalein, wogonoside, and wogonin which have a rarely occurring unsubstituted B-ring (**Figure 1**) (Zhao et al., 2016; Zhao et al., 2019).

Among the many properties of Sb’s main flavonoids, their potent cytostatic and cytotoxic effects observed both *in vitro* and *in vivo* are worth noting (Gu et al., 2022; Shah et al., 2022). They were found to be effective against various neoplasms, such as myeloma, prostate, colon, and lung cancer (Zhao et al., 2019; Sui et al., 2021; Gu et al., 2022). Wogonoside is a promising cytotoxic agent, especially in the treatment of non-small cell lung cancer (NSCLC), which is one of the most difficult lung cancer types to cure (Luo et al., 2018). Its aglycone, wogonin, was found to enhance the cytotoxic effect of other chemotherapeutics, e.g., oxaliplatin (Hong et al., 2018; Gu et al., 2022). All four main Sb flavonoids strengthen the immune system and have potent anti-inflammatory and anti-allergic properties (Liao et al., 2021). Wogonin and baicalein can be used in the prevention and treatment of mast cell-mediated asthma (Zhao et al., 2019). Baicalein promotes neuron recovery and could be used in the treatment of memory loss and neurodegenerative disorders such as Alzheimer’s and Parkinson’s diseases (Aliev et al., 2011; Jeong et al., 2011; Zhao et al., 2019). Wogonin exhibits anxiolytic and anticonvulsant effects (Zhao et al., 2019; Huynh et al., 2020). Wogonin can not only be a new therapeutic agent on its own but can also serve as a good model in the development of new, selective, and reversible MAO-A inhibitors, i.e., antidepressants (Lee et al., 2017). In Traditional Chinese Medicine, Sb has been used in the treatment of hepatic disorders since ancient times and its compounds (especially baicalin) were recently proven to have potent antifibrotic and hepatoprotective properties (Wang et al., 2018; Zhao et al., 2019; Liao et al., 2021). Sb antibacterial activity is not limited to common pathogens; it also inhibits the growth of oral bacteria and can prevent dental caries (Zhao et al., 2019; Gościniak et al., 2021). Baicalin’s inhibitory activity toward methicillin- and vancomycin-resistant *Staphylococcus aureus* (MRSA and VRSA) makes it a promising candidate for new drugs able to combat infections caused by these pathogens, which are very difficult to cure using conventional antibiotics (Zhang et al., 2020). Sb extract also has antiviral properties and was shown to enhance innate antiviral immunity (Zhao et al., 2019; Liao et al., 2021). Baicalin and baicalein were also found to have potent anticoronaviral activity *in vitro* by inhibiting the SARS-CoV-2 3CLpro enzyme. Further *in vivo* and clinical studies of this effect may lead to the development of new drugs used in the treatment of COVID-19 (Su et al., 2020; Pei et al., 2022). Sb flavonoids have the ability to scavenge free radicals and protect against lipid peroxidation (Zhao et al., 2019). Moreover, baicalein can lower the production of pro-oxidative compounds by decreasing the expression of iNOS and COX-2 genes (Aliev et al., 2011). Baicalin and baicalein could be utilized in the therapy of craniocerebral injuries associated with free radical attacks (Zhao et al., 2019) and show promise in the development of new antidiabetic drugs since radical-induced oxidative stress and the decrease of intrinsic antioxidant activity are the main causes of vascular complications in diabetics (Zhao et al., 2019). The antioxidant, anti-inflammatory, antidepressant, and neuroprotective properties described above contribute to the widespread classification of this herbal drug among natural adaptogens. Constituents of phytopreparations demonstrating adaptogenic properties often include phenolic compounds such as flavonoids, phenylpropanoid glycosides, hydroxycinnamic acids, and lignans. For example, the total flavonoids from *Scutellaria comosa* Juz. that are comparably rich in flavonoids to *S.baicalensis* can increase the adaptive capabilities of the body, relieving physical fatigue and activating the recovery process (Ergasheva et al., 2023). Therefore, we believe that using the combined approach reported here, of electrostimulation with aeroponics to influence the content of these highly active compounds paired with increased gain in root biomass and growth speed, would contribute to more precisely controlled cultivation and the obtainment of profitable yields of high and predictable quality of raw material for pharmaceutical applications, including the development of new phytopreparations in the future.

## Materials and methods

### Materials

All solvents (methanol and acetonitrile) used for this work were of analytical grade and purchased from Merck (Darmstadt, Germany). Ultrapure water was obtained from the Milli-Q^®^ Simplicity 185 system (Millipore Corp., Billerica MA). MS-grade formic acid (FA) and KCl were also used.

### Cultivation

Sb was grown from seeds acquired from the certified collection of the Botanical Garden of Medicinal Plants at the Medical University of Wroclaw. They were surface sterilized in NaClO (3% w/v) for five mins, then rinsed four times with sterile water, and sown on wetted filter paper in 100x20 mm glass Petri dishes for germination. Seeds were stratified at 4°C for four days and then placed in a growth chamber at 22°C, with a 16/8 h light/dark photoperiod. After sprouting, when the primary root length was approximately 2cm, seedlings were transferred to aeroponic plastic pots (10cm in diameter) together with a sponge in sterile conditions and moved to X-stream Aero aeroponic systems filled with water acquired in the process of reverse osmosis (Power Grow 500 reverse osmosis filter). Following this, 20 mL (5mL per 1L of water) of ROOT!T First Feed nutritional medium (composition: 1.7% NO_3_, 0.1% NH_4_, 2.2% K_2_O, 1.0% P_2_O_5_, 1.9% CaO, 0.9% MgO, 0.6% SO_3_, 0.04% Fe, and 0.007% Zn) was then added and replenished every week. Plants were kept at 20-24°C and exposed to white light for 8h/day. Six-week-old plantlets with well-developed roots and shoots were then subjected to electroporation. After treatment, the plantlets were washed with deionized water and reintroduced to the aeroponic systems inside a Growbox Dark Room R2.50 tent illuminated with mixed red (610 – 600 nm) and blue (450 – 470 nm) light generated by Phytoled GX-300 Phytolite Full Cycle panels for 8h/day. Conditions inside the tent (T = 25-28°C; humidity = 40-50%) were maintained using a Can Fan (100 mm) and filters (300-330 m^3^/h) ventilation system. Plants were left to grow there until reaching the age of 3.5 months. During this period, the nutritional medium was refilled every week and plant development was recorded every two weeks. Photographs of plants were taken after putting all specimens of the given subgroup on a 1x1 cm grid. Each specimen was later measured digitally using ImageJ and the increases in root and shoot lengths over a period of four weeks were calculated.

### Electroporation

The experiment was conducted on six-week-old plantlets. To generate the impulses, a BTX ECM 830 Square Wave Electroporation System was used. During impulse administration, each individual plantlet was put inside a BTX Electroporation Cuvette Plus (4mm gap between electrodes) filled with 1 mL of conductive medium (40 mM KCl, pH = 6.2; conductivity = 4.6 mS/cm). The experiment was divided into five groups (**Table 1**) by the strength of the applied electrical field (E [kV/cm]), which were then further divided into three subgroups by duration (t [μs]) and the number of impulses (N) delivered. Control groups were treated the same way, except for the application of any electric field. Each studied subgroup and the control consisted of five Sb individuals. Each time, after delivering the appropriate impulses, the conductive medium was changed to a fresh portion.

**Table 1:** Parameters of electroporation.

Parameter	Group 1			Group 2			Group 3			Group 4			Group 5			Control
**E [kV/cm]**	1.25			1.75			2.25			3			5			–
**t [μs]**	10	25	100	10	25	100	10	25	100	10	25	100	10	25	100	–
**N**	100	40	10	100	40	10	100	40	10	100	40	10	100	40	10	–

E - electric field strength, t - duration of impulse, N - number of impulses delivered, - means no applied electric field (control plants).

### Extraction and LC-MS analysis

All 3.5 months old plants (PEF treated and control) were removed from aeroponics, the roots were dissected, weighed, and then dried at room temperature for two weeks. After this process, the roots were weighed again on the same scale and then minced in a mortar. Rootlets were separated from the main root in accordance with European Pharmacopoeia guidelines, with the use of a sieve. Plants from the E = 1.75 kV/cm, t = 25 μs, N = 40 subgroup mainly produced rootlets and the main root mass was insufficient to prepare an extract from, so they were discarded altogether. Three biological samples, A, B, and C, each containing 0.1g of dried and minced Sb main roots from each subgroup, were prepared. The material was then moved to 1.5 mL Eppendorf vials where 1 mL of 80% MeOH with 0.1% formic acid (FA) was applied as an extraction solvent. Ultrasound-assisted extraction was conducted for 30 minutes at room temperature (five-second pauses between 20 seconds of ultrasounds delivered in an ultrasonic bath). Then, the samples were centrifuged (5000 rpm for five mins) and the supernatant was collected. This process was repeated three times, and the extracts were combined and dried under nitrogen. Finally, the dry extracts were dissolved in 1 mL of 80% MeOH with 0.1% FA. This solution was then again subjected to ultrasound until complete dissolution and filtered (0.45 µm HPLC filter) before LC-MS analysis. Standard stock solutions, baicalein (CAS Number 491-67-8), baicalin (CAS Number 21967-41-9), wogonin (CAS Number 632-85-9), and wogonoside (CAS Number 51059-44-0), purchased from Sigma-Aldrich (St. Louis, USA) and kept frozen until analysis, were also prepared by dissolution in 80% MeOH resulting in a range of concentrations for each individual standard, (0.0234-0.75 mg/mL), (0.0266-0.85 mg/mL), (0.025-0.80 mg/mL), and (0.025-0.80 mg/mL), respectively, to prepare calibration curves based on seven concentration points. UV chromatogram was recorded at 280 nm.

Sb extract samples were analyzed using UHPLC-HR-MS using a Dionex UltiMate 3000RS (Thermo Scientific, Darmstadt, Germany) system interfaced with a high-resolution quadrupole time-of-flight mass spectrometer (HR/Q-TOF/MS, Compact, Bruker Daltonik GmbH, Bremen, Germany). Separation was performed using a Kinetex C18 column (2.1 × 100 mm, 2.6 μm, Phenomenex, USA) maintained at 30°C. The mobile phase consisted of A (0.1% FA acid in Milli-Q water, v/v) and B (0.1% FA in acetonitrile, v/v) at a flow rate of 0.3 mL/min. The gradient elution was 2% B from 0 to one min with a 0.3 min calibration segment, and the concentration of B was then increased to 60% from one to 20 min. The column was eluted with this concentration of solvent B for four mins and then re-equilibrated for 0.3 min and back to 2% B for the next 3.7 min. The samples were kept at 15°C in the autosampler. The injection volume was 5.0 μL. The mass spectrometer operated in both positive and negative electrospray ionization (ESI) modes. The ion source parameters were as follows: the capillary voltage was set at 4.0 kV, corona voltage was set at 8.0 kV, nebulizer pressure was 2.5 bar, dry gas flow was 1.5 L/min, dry temperature was 200°C, and vaporizer temperature was 320°C. The mass scan range was from 100 to 1200 *m/z* with a 5 Hz spectral acquisition rate. MS/MS spectra were acquired in a data-dependent manner, whereby precursor ions (maximum two) from each scan were subjected to collision-induced fragmentation if their absolute intensity exceeded 1800 counts. Variable collision energy ranging from 15 to 40 eV was used depending on the *m/z* of the selected precursor ions. Internal calibration used tuning mix diluted 1:4 (vol/vol) with 50% 2-propanol and was introduced to the ion source *via* a 20 μL loop at the beginning of each analysis using a six-port valve. Data were collected and processed by DataAnalysis 4.3 (Bruker Daltonik GmbH, Bremen, Germany). All analyses were performed in triplicate.

The DataAnalysis 4.3 software (Bruker Daltonics GmbH, Bremen, Germany) provides a ranking according to the best fit of measured and theoretical isotopic patterns within a specific mass accuracy window. The quality of the isotopic fit was expressed by the mSigma-value. The SmartFormula3D matched peaks were sent to the MetFrag website in silico fragmentation for computer-assisted identification of metabolite mass spectra. Additionally, internet databases were used to search for the structural identity of the metabolites and these included: the Human Metabolome database (http://www.hmdb.ca/), the BiGG database (http://bigg.ucsd.edu/), PubChem (http://pubchem.ncbi.nlm.nih.gov/), MassBank (http://www.massbank.jp), KEGG (www.genome.jp), and Metlin (http://metlin.scripps.edu), as well as being supported with MS/MS information recovered from the literature (Xu et al., 2018).

### Statistical analysis

One- and two-way ANOVA and Student’s t-test analyses were conducted using GraphPad Prism 9, and Simca 16.0 was used for PCA and OPLS analyses.

## Results and discussion

### Plant development and root biomass

**Figure 2:**
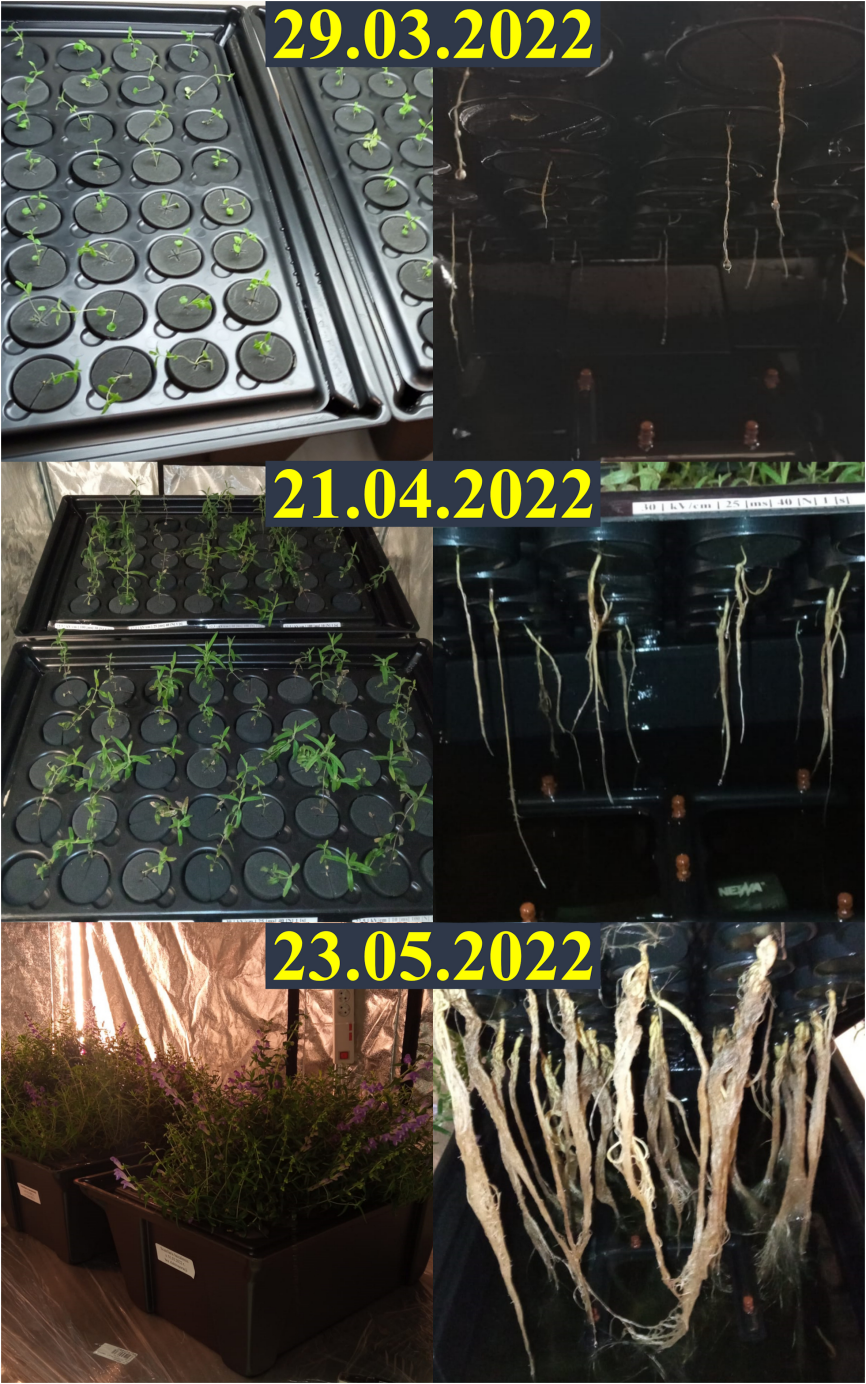
Overall monthly development of S. baicalensis shoots and roots.

**Figure 3:**
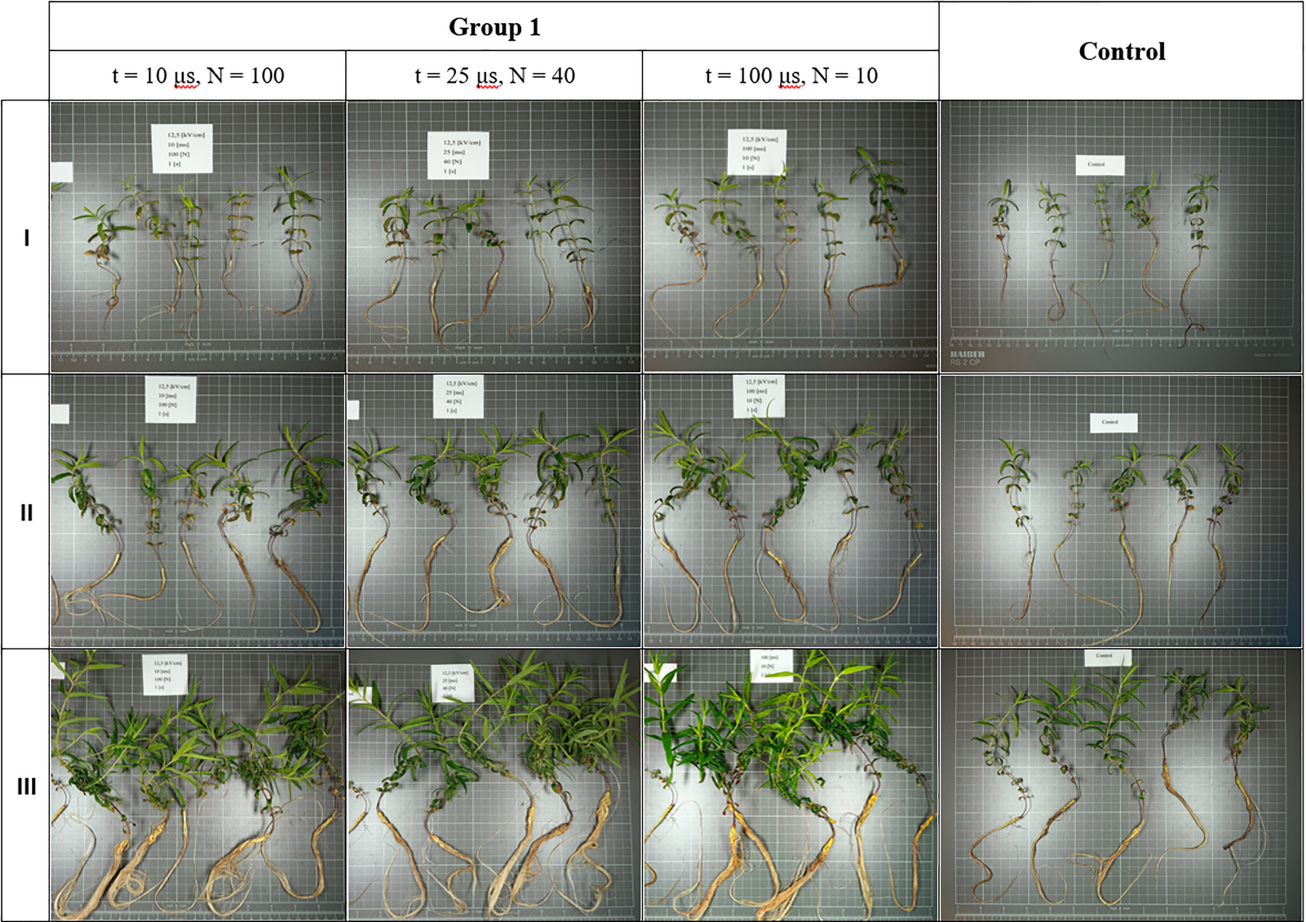
Growth and development of S. baicalensis in two-week intervals shown on a 1cm x 1cm grid (row I – two weeks after treatment, row II – four weeks after, row III – six weeks after).

All treated plants exhibited relatively similar growth and development rates of both roots and shoots (**Figure 2**), but most individuals grew larger in size than those from the control group (**Supplementary Figures 1-4**). The average increase in root and shoot length per plant over a period of four weeks in all treated subgroups (an increase in shoot length from 6.54 cm in group five up to 11.87 cm in group two; an increase in root length from 10.82 cm in group five up to 16.85 cm in group one) was higher than for control (5.66 cm increase in shoot length and 4.86 cm increase in root length). In groups two and three, plants from the medium intensity subgroup (t = 25 μs, N = 40) were in visibly worse condition (the former was then discarded altogether, and the latter showed average increases in shoot length of 5.99 cm and root length of 9.51 cm, and was one of the slowest growing cultures, although still surpassing controls). This is evident in later comparisons of fresh and dried root masses derived from them. Plants subjected to 5 kV/cm electric field strength (group five) grew at the lowest rate, but even under such harsh treatment conditions, some plants from this group still managed to grow as large or even larger than those from control groups. This is true for all other treatment parameters; however, the growth-promoting effect of electroporation was most evident in group one (**Figure 3**). This suggests that in our case, an electric field strength equal to (and/or lower than) 1.25 kV/cm was optimal for achieving intensified growth of *S. baicalensis* in aeroponics, but further studies are necessary. The parameters of PEF treatment have a significant influence on the electroporation process; increasing them can result in stronger cell permeabilization (Donsì et al., 2010). The steady increase from group one to five along with the decrease in plant size and growth rate reflected a well-described phenomenon: that, after exceeding optimal treatment parameters (in this case, group one), growth stimulation is increasingly masked by injury (Eing et al., 2009). The survival and development of plants in group five highlighted the great positive impact of cultivation in aeroponics. This technology has proved to be very efficient and successful in plant cultivation, which is the most frequently highlighted advantage of this horticulture practice (Lakhiar et al., 2018; Komosa et al., 2020).

**Figure 4:**
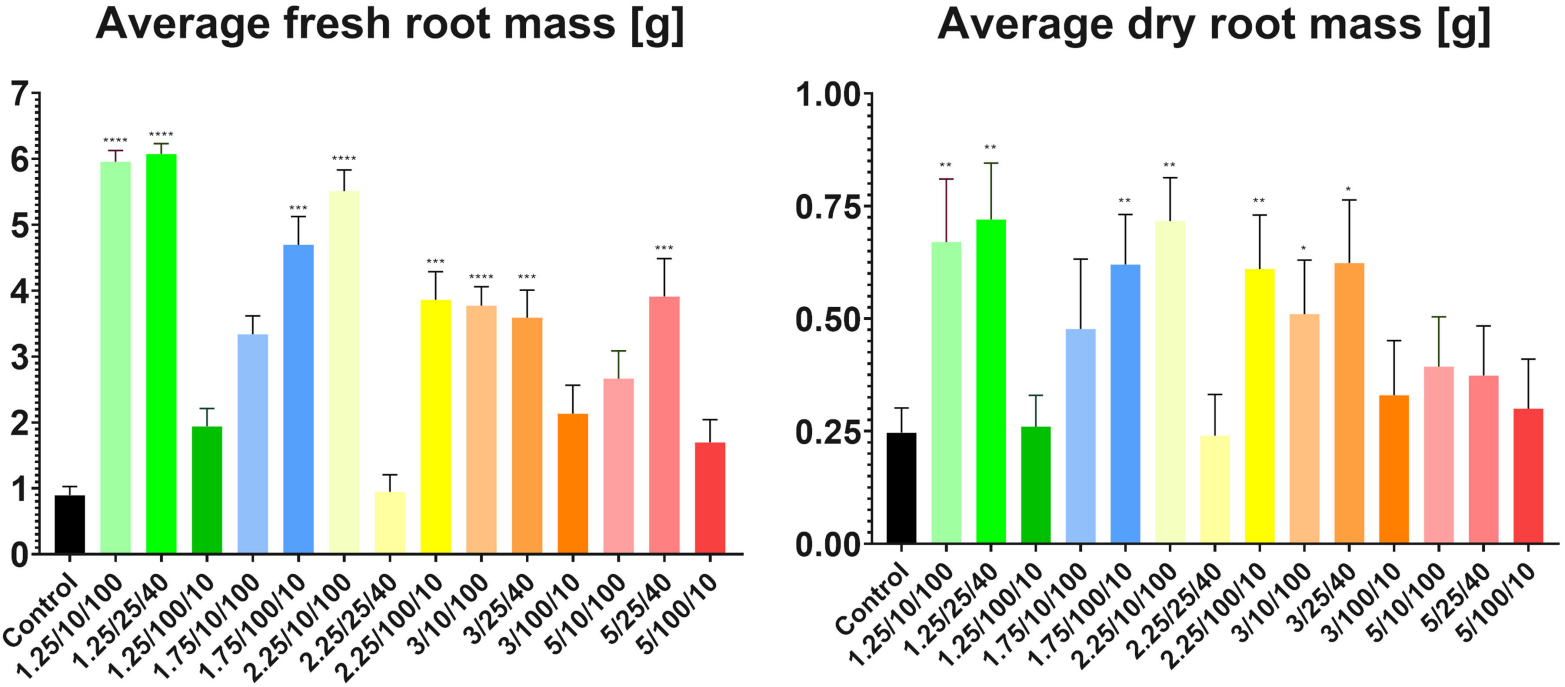
Measurements of average fresh and dried root masses per plant. Asterisks highlight groups showing the most statistically significant differences in weight in comparison to controls (**** = P<0.0001 and decreases in their number correlate to an increase in P value) found after conducting Student’s t-test. Parameters shown on the X-axis are presented in the following order: E/t/N.

The lowest average fresh mass (0.89g) was found in the control group, and the lowest average dried mass (0.24g) was found in the medium intensity subgroup from group three (E = 2.25 kV/cm, t = 25 μs, N = 40) (**Figure 4**). Both the highest average fresh (6.07g) and average dried (0.72g) masses were found in the medium intensity subgroup from group one (E = 1.25 kV/cm, t = 25 μs, N = 40). This observation confirmed that, after proper adjustment of the parameters, electroporation can significantly increase plant growth and mass gain (Eing et al., 2009).

### Flowering

**Figure 5:**
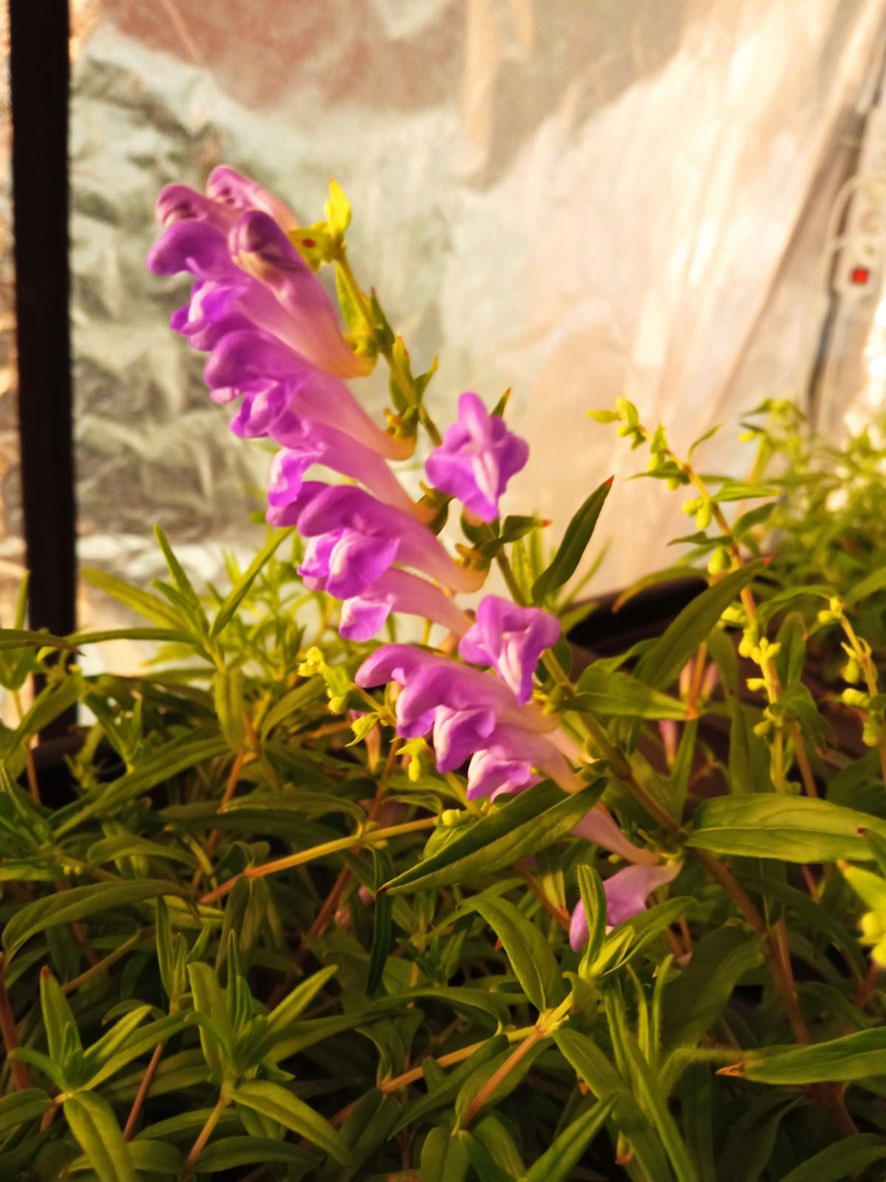
One of the first plants to blossom shown in full bloom from E = 1.25 kV/cm, t = 100 μs, N = 10 subgroup.

The first blossoming (**Figure 5**) was observed 33 days after treatment (E = 1.25 kV/cm, t = 100 μs, N = 10). Two days later (35 days), single plants from other subgroups (E = 1.75 kV/cm, t = 100 μs, N = 10 and E = 2.25 kV/cm t = 10 μs, N = 100) also blossomed. In the following days, flowering was observed in all other treated groups and lastly, 41 days after treatment, plants from the control group also blossomed. The illumination regime used in growth chambers is known to facilitate flowering (blue light) and intensify photosynthesis (red light) (Yoshida et al., 2016) but that the control group was the last to produce flowers, more than a week after the first such observation, hints at the possible anthesis-promoting effect of electroporation. A possible mechanism may depend on the reaction of developmental signaling pathways to oxidative stress caused by electroporation (Sabri et al., 1996). This phenomenon is much better described in mammalian cells, where it was clearly shown that electroporation induced the generation of ROS inside the cells (Szlasa et al., 2020; Batista Napotnik et al., 2021). Synthesis of various phytochemicals that increase antioxidant capacity, such as flavonoids, is a typical response to a similar stimulus – high salinity – which is also documented to cause oxidative stress to the roots (AbdElgawad et al., 2016; Shah and Smith, 2020). Flavonoids have also been documented to play a significant role in the sexual reproduction of plants (Ingrosso et al., 2011), and intensifying their biosynthesis might speed up the process of plant maturation and therefore blossoming.

### Phytochemical analysis

**Figure 6:**
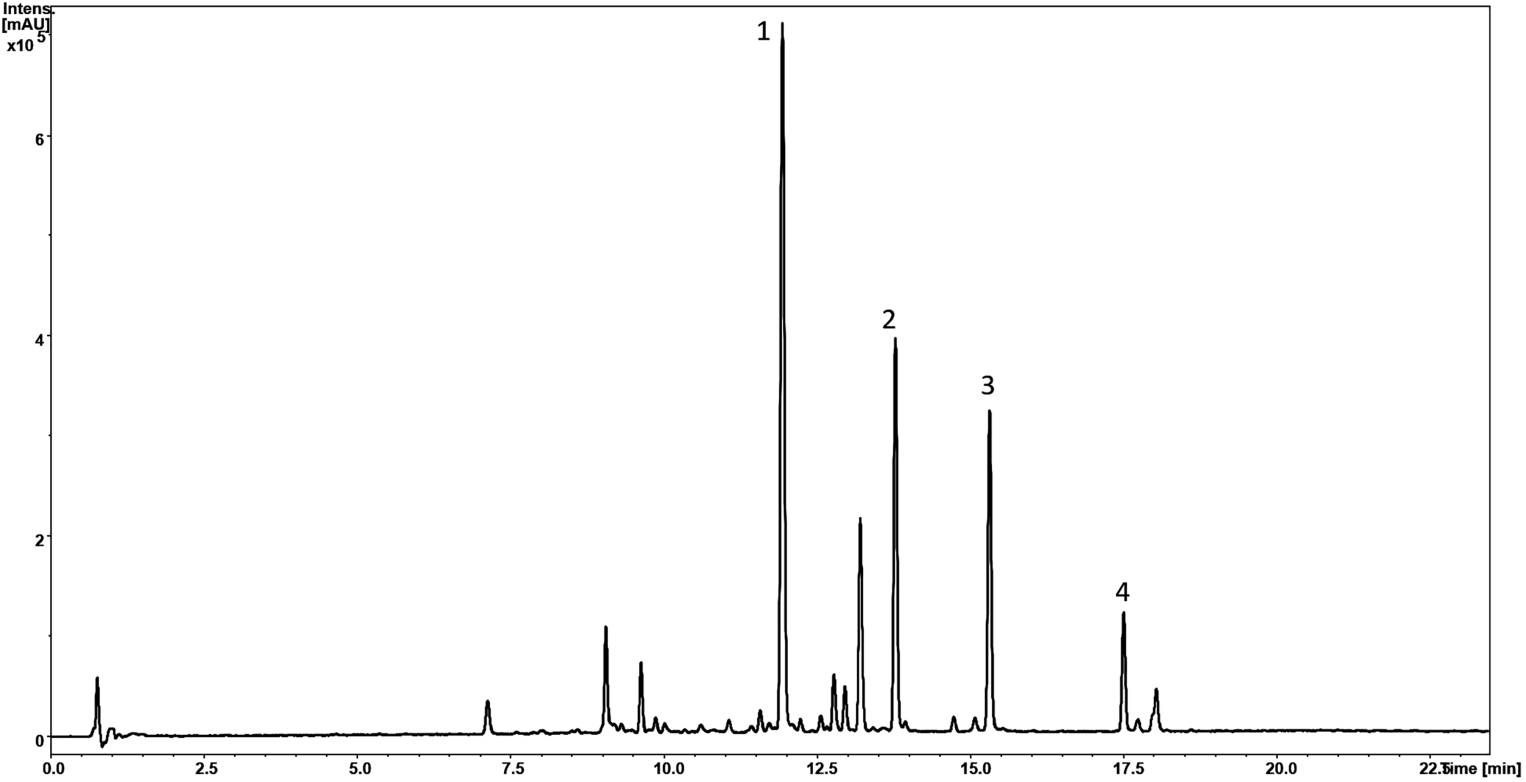
HPLC-MS chromatogram (UV 280 nm) of S. baicalensis root methanolic extract (Control) with marked peaks corresponding to baicalin (1), wogonoside (2), baicalein (3), and wogonin (4) concentrations.

Phytochemical profiling *via* LC-MS analysis of Sb root methanol extract was conducted (**Table 2**; **Figure 6**). Most of the detected compounds were confirmed according to the available literature (Wang et al., 2018; Zhao et al., 2019). They also had much higher baicalin and wogonoside content than other organs of this plant, especially when at the full bloom stage. This analysis was conducted on plants approximately 3.5 months old (exactly 113 days after sowing) since around this time baicalin and wogonoside contents in roots of *S. baicalensis* are documented to be at their highest (Xu et al., 2018). It has been shown that the concentration of glucuronides is much higher than that of aglycones and also that baicalin is a dominant compound (Kosakowska, 2017). This trend was also evident in our study. Islam et al. (2011) established that roots of three-month-old Sb cultivated on agar medium in a greenhouse contained the following main flavonoid concentrations: baicalin – 25.41 µg/mg, wogonoside – 12.63 µg/mg, baicalein – 1.04 µg/mg, and wogonin – 6.71 µg/mg. Levels of these compounds in the control group presented in this study were lower, but comparable in the case of glucuronides (baicalin – 20.67 µg/mg and wogonoside – 8.90 µg/mg) and baicalein – 0.60 µg/mg. However, wogonin content was significantly lower (0.25 µg/mg). These differences might be caused by the different cultivation conditions.

**Table 2:** *Scutellariae baicalensis radix*.

Compounds	Rt(min)	UV	m/z[M-H]-	Error [ppm]	formula	MS^2^ ion
tryptophan	4.50	220, 280	203.00	0.4	C_11_H_12_N_2_O_2_	116(100), 142(47)
3,5,7,2’,6’-pentahydroxyflavanone	7.60	210, 290	303.0510	0.4	C_15_H_12_O_7_	125(100), 177(65), 149(65), 193(27), 165(27), 217, 285
Apigenin 7-O-[β-D-apiosyl-(1->2)-β-D-glucoside]	8.00	220, 272, 335	563.1397	1.60	C_26_H_28_O_14_	353(100), 383(74), 443(18), 325(13), 473(13)
5,7,2’,6’-Tetrahydroxyflavanone 2-O-β-D-glucoside	8.25	220, 290	449.1079	2.20	C_21_H_22_O_11_	125(100), 177, 287
6-C-Glucose-8-C-rhamnose-chrysin	8.40	220, 273, 315	577.1552	0.2	C_27_H_30_O_14_	337(100), 367(59), 309(13)
3,5,7,2’,6’-Pentahydroxy flavone	8.60	220	301.0345	0.80	C_15_H_10_O_7_	149(100), 151(65), 175(25), 125(4)
6-C-Arabinose-8-C-glucose-glucose-chrysin	8.70	220	709.1960	1.40	C_32_H_38_O_18_	443(100), 243
6-C-Arabinose-8-C-glucose-chrysin	9.10	215, 275, 315	547.1447	0.1	C_26_H_28_O_13_	337(100), 367(83), 457(13), 309(12), 427(10)
Dihydroscutellarein-7-O-β-D-glucuronide	9.15	215, 275	463.0875	2.30	C_21_H_20_O_12_	287(100), 166(30), 181(15)
Scutellarein-7-O-β-D-glucuronide (Scutellarin)	9.30	220, 280, 330	461.0712	0.14	C_21_H_18_O_12_	285
6-C-Glucose-8-C-arabinose-chrysin	9.60	215, 275, 315	547.1448	0.1	C_26_H_28_O_13_	337(100), 367(68), 427(44), 457(27)
5,7,2’-Trihydroxy-6-methoxy flavone-7-O-β-D-glucuronide	11.10	215, 270, 337	475.0873	1.80	C_22_H_20_O_12_	284(100), 299(35), 285(13.7), 300(5), 165(2,6)
5,7,2’,5’-Tetrahydroxy-8,6’-dimethoxy flavone	11.60	215, 265, 338	345.0607	2.50	C_17_H_14_O_8_	315(100), 330(15), 316(14), 164(6.5)
Baicalin^a^	11.85	225, 280, 315	445.0771	1.50	C_21_H_18_O_11_	269(100), 251(8)
Dihydrobaicalin	12.60	215, 290	447.0929	0.80	C_21_H_20_O_11_	271(100), 243(58), 253(13), 244(97)
5,7-Dihydroxy-6-methoxy flavone-7-O-β-D-glucoside	12.70	220, 280	445.1137	0.80	C_22_H_22_O_10_	267(100), 283(5)
Norwogonin-7-O-β-D-glucuronide	12.85	215, 280, 360	445.0771	1.50	C_21_H_18_O_11_	269(100)
5,6,7-Trihydroxy-8-methoxy flavone-7-O-β-D-glucuronide	13.00	215, 280	475.0879	0.80	C_22_H_20_O_12_	284(100), 299(16.7), 285(13), 300, 283
Oroxylin A 7-O-β-D-glucuronide	13.20	210, 270, 310	459.0928	-1.20	C_22_H_22_O_11_	268(100), 283(16), 269(13)
chrysin-glucuronide	13.30	270, 310	429.0821	1.10	C_21_H_18_O_10_	253(100)
5,7,8-Trihydroxy-6-methoxyflavone-7-O-β-D-glucuronide	13.40	210, 290	475.0879	0.70	C_22_H_22_O_12_	284(100), 299(33), 285(13), 300(4.5)
Wogonoside^a^	13.80	220, 275	459.0929	0.90	C_22_H_22_O_11_	268(100), 283(15), 269(13), 284(2.2)
5,7-Dihydroxy-6,8-dimethoxyflavone-7-O-β-D-glucuronide	13.90	220, 280	489.1035	0.70	C_23_H_22_O_12_	298(100), 283(45), 299(14), 313(13)
norwogonin	14.70	220, 280	269.0453	0.40	C_15_H_10_O_5_	197(100), 171(40), 213(31)
5,6,7-Trihydroxy-4-methoxyflavone	15.10	220, 280	299.0561	-0.10	C_16_H_12_O_6_	284(100), 181(14), 153(14), 285(13), 200(11)
Baicalein^a^	15.40	215, 275, 320	269.0457	0.40	C_15_H_10_O_5_	223(24), 241(20), 195(17), 169(15), 197(12), 251(11), 271(8)
Wogonin^a^	17.50	210, 275	283.0616	1.60	C_16_H_12_O_5_	268(100), 163(23), 184, 239
chrysin	17.80	220,270,310	253.0510	-1.40	C_15_H_10_O_4_	209, 166, 143
5,2’-Dihydroxy-6,7,8,6’-tetramethoxyflavone (Skullcapflavone II)	17.95	220, 270, 320	373.0934	-1.40	C_19_H_18_O_8_	343(100), 328(64), 300(24), 194(10), 358
oroxylin A	18.05	215, 270, 320	283.0613	-0.50	C_16_H_12_O_5_	268(100), 165(8.8), 184(7), 239
4’,5-Dihydroxy-6,7,8-trimethoxyflavone	18.60	220, 280	343.0821	0.60	C_18_H_16_O_7_	313(100), 298(67), 270(18.6), 299(9), 328, 194

Identification based on isolated standards^a^ or CompoundCcrowler 3.1 program (Bruker Daltonoics Darmstad) for searching databases, supported with the MetFrag database and appropriate literature.

**Figure 7:**
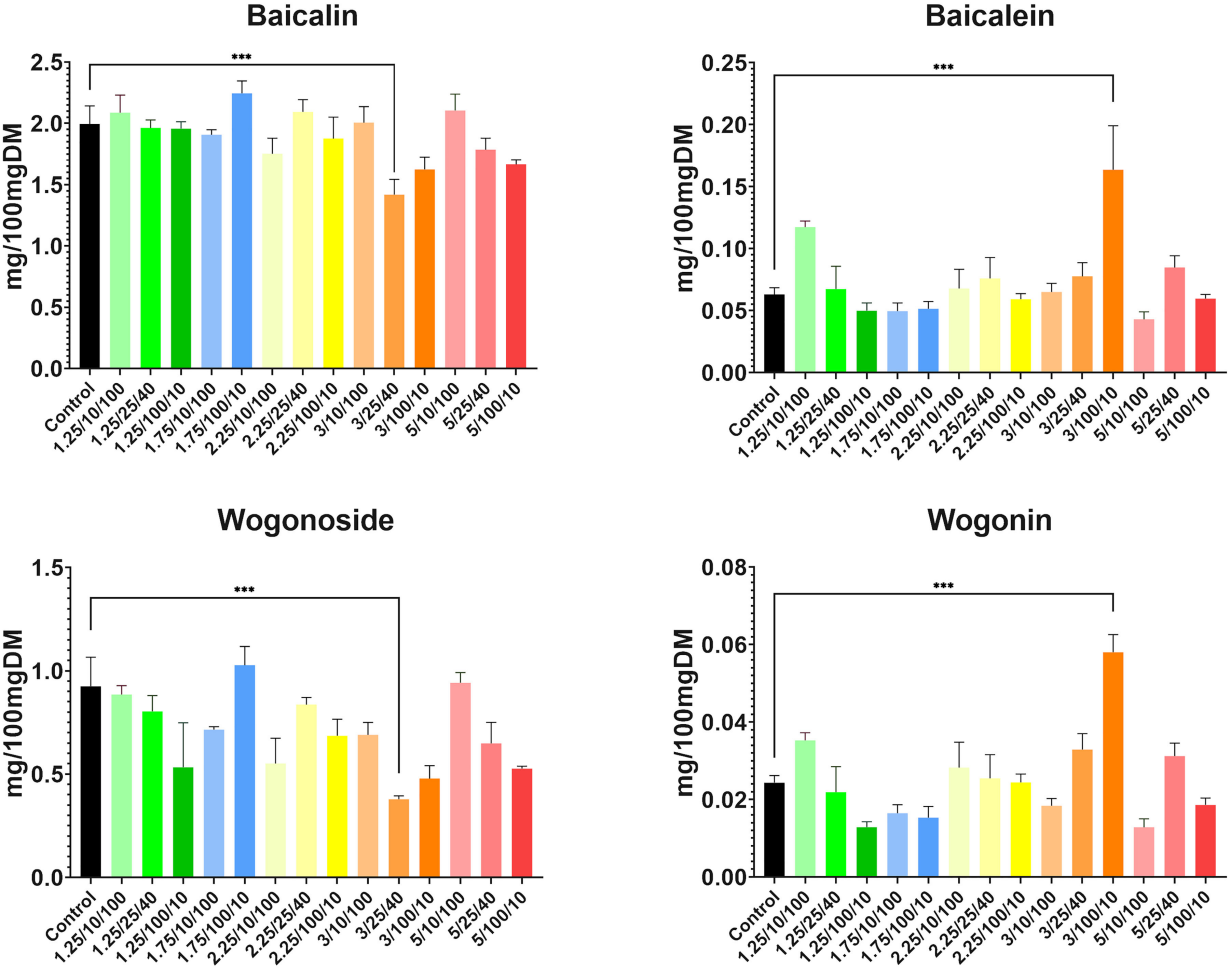
Concentrations of four main compounds of interest yielded from each subgroup with the largest statistical differences (*** indicates P<0.0001) between treated subgroups and controls, highlighted by asterisks found after conducting a two-way ANOVA analysis. Parameters shown on the X-axis are presented in the following order: E/t/N. DM stands for dried mass.

As for flavonoid concentrations, the results were also very promising and the role of electroporation as an abiotic stressor that stimulates plant metabolism was highlighted. Concentrations of flavones were higher in comparison to control samples in at least half of the treated plants (**Figure 7**). An increase in baicalein (from 0.0609 mg/100mg DM up to 0.1518 mg/100mg DM across 9 subgroups, compared to 0.0602 mg/100mg DM yielded from control) and wogonin (from 0.0252 mg/100mg DM up to 0.0568 mg/100mg DM across 7 subgroups, compared to 0.0242 mg/100mgDM in control) content was observed. Concentrations of both compounds increased approximately 2.5-fold in the highest intensity subgroup of group four (E = 3 kV/cm, t = 100 μs, N = 10). This suggested that these parameters are optimal for increasing aglycone content in the roots of *S. baicalensis* with great success.

However, the increase in glucuronide content was much less significant, suggesting an adaptational response to stress conditions. Baicalin concentration grew in five subgroups (from 2.120 mg/100mgDM up to 2.203 mg/100mgDM, compared to 2.067 mg/100mgDM yielded from controls) while wogonoside content was increased only in three treated subgroups (from 0.8976 mg/100mgDM up to 1.016 mg/100mgDM compared to 0.8895 mg/100mgDM yielded from controls). In both cases, this increase was most significant in the highest intensity subgroup from group two (E = 1.75 kV/cm, t = 100 μs, N = 10); however, it was not as spectacular as in the case of aglycones. Also, in the case of the latter compound, most of the treatments resulted in a significant decrease in its concentration whereas, in the case of baicalin, the content remained similar to the control but decreased in six subgroups.

Therefore, the alterations in the phytochemical profile of the major flavones were more pronounced than the increase in their summed content. The shift towards aglycons may have resulted from the increased hydrolysis of the conjugated forms as part of the stress response or/and suppressed expression or activity of glycosylating enzymes. This phenomenon has been widely reported in many plant species already, but there is no clear proof that aglycones appear only because of stress responses (Behr et al., 2020). Baicalin and wogonoside have weaker antioxidant properties than their aglycone counterparts (Woźniak et al., 2015) which may explain their adaptive role in response to oxidative stress. To clarify this, an extensive study would be necessary involving the molecular level of gene expression (such as glucuronosyltransferases, recently defined by Pei et al., 2022) and the activity of endogenous enzymes accompanied by metabolic flux observations. In the present study, we can hypothesize that electroporation acted as a complex stimulus for a variety of physiological and molecular mechanisms.

In the available literature, we were unable to find similar research using PEF as a direct stimulation of flavonoid production in *Sb* or closely related plants. However, such stimulation in the cell suspension culture of *Vitis vinifera* L. resulted in a 1.7-fold increase in anthocyanin production under the direct influence of PEF stimulation on the 14^th^ day of culturing (1.42 mg/g DW) in anthocyanin content compared to control cells. (Saw et al., 2012) In *Triticum aestivum* L. (wheat) seedlings, PEF treatment at 6 kV/cm at 50 pulses increased the water uptake, germination of seeds, and growth parameters. A significant increase in total phenolic contents, anti-radical activity, chlorophylls, carotenoids, soluble proteins, minerals, and amino acids in PEF‐treated plants was observed. The results indicated that PEF may effectively stimulate the growth of the wheat kernels and positively affect their metabolism (Ahmed et al., 2020).

Furthermore, the application of an electric field can also significantly elicit the production of phenolic antioxidants in two cultivars of kale (*Brassica oleracea* convar. *acephala* (DC.) Alef.) (Lee and Oh, 2021). Plants grown under a 50 mA electric current contained 72% more calcium, 57% more total phenolic compounds, and had a 70% greater antioxidant capacity than the controls. In yet another study, sub-lethal levels of an electric current led to the increased content of specialized metabolites in different transgenic and non-transgenic plant tissues (Kaimoyo et al., 2008). Seedlings, intact roots, or cell suspension cultures of *A. thaliana*, fenugreek (*Trigonella foenum-graecum* L.), barrel medic (*Medicago truncatula* Gaertn.), red clover (*Trifolium pretense* L.), and chickpea (*Cicer arietinum* L.) produced increased levels of metabolites in response to electro-elicitation. Based on these reports, electric currents would appear to be a general elicitor of plant-specialized metabolites and have a great potential for use in both basic and applied plant sciences.

**Figure 8:**
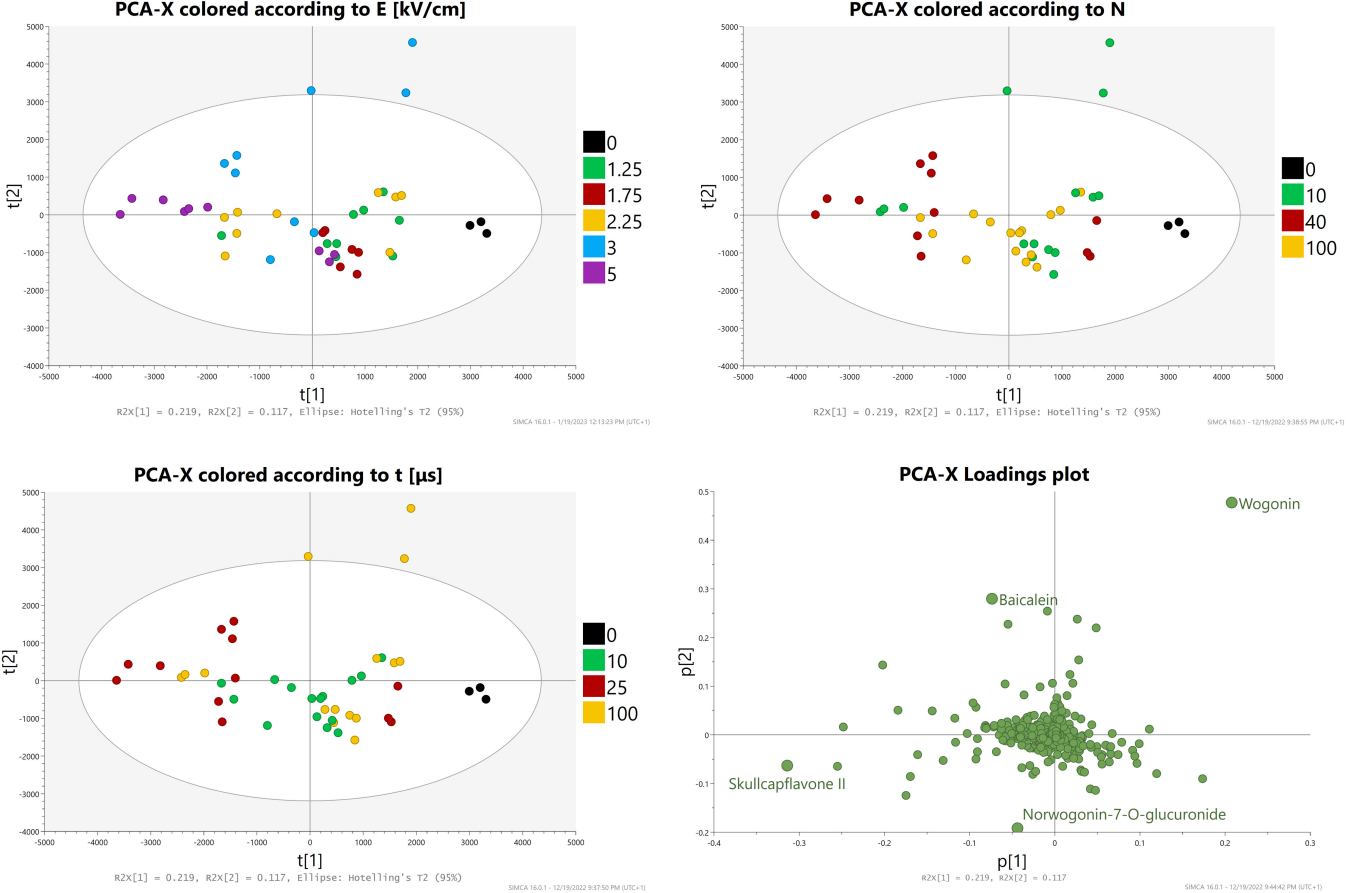
PCA-X analysis - Score and loading plots.

PCA-X analysis with Pareto scaling was conducted (**Figure 8**). The score plots revealed three outliers, all of them being repetitions A, B, and C of the E = 3 kV/cm, t = 100 μs, N = 10 subgroup which yielded the highest aglycone concentrations. Samples from the control and group two were closely related. The loading plots revealed the compounds most significant for this model, which were baicalein, wogonin, norwogonin-7-O-glucuronide, and skullcapflavone II.

**Figure 9:**
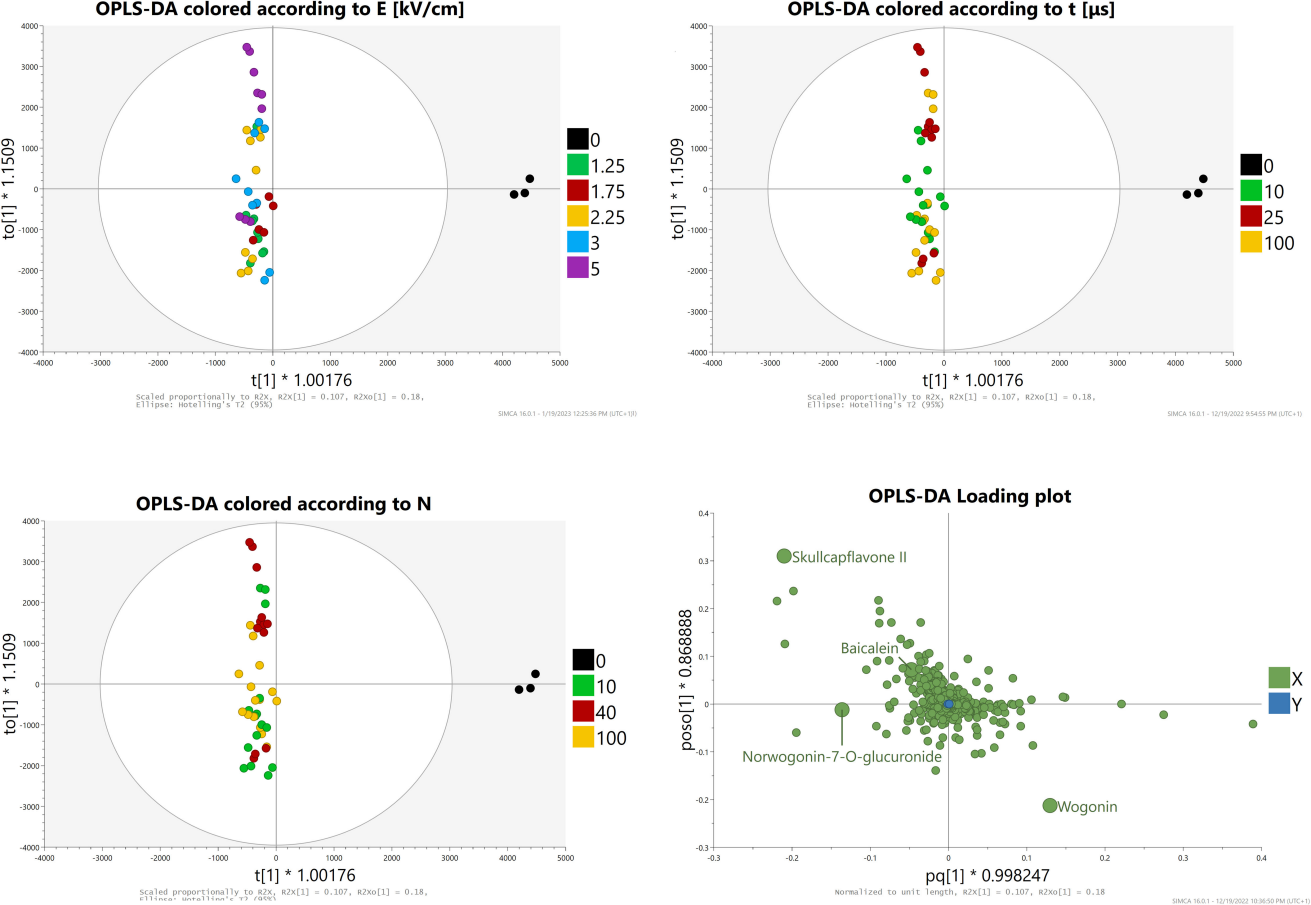
OPLS-DA analysis Score and loading plots.

OPLS-DA analysis with Pareto scaling was then conducted (**Figure 9**). The score plots showed a clear separation between control and treated groups and highlighted similarities (especially between groups one and two) and differences between the provided samples. Groups four and five were both scattered across the Y-axis which hinted at a great differentiation of results depending on the duration and amount of impulses applied (i.e., between subgroups). The loading plots showed that, in comparison to PCA-X analysis, baicalein and norwogonin-7-O-glucronide lost much of their statistical significance, while wogonin and skullcapflavone II retained it.

In summary, these two analyses showed that treatment applied in the highest intensity subgroup from group four led to a statistically significant increase in the concentrations of wogonin and very likely baicalein.

## Conclusions

We conclude that electroporation combined with aeroponics can result in increased flavonoid content after careful adjustment of EP parameters. The concentration of aglycones was drastically increased (approx. 2.5-fold) under following treatment conditions: E = 3 kV/cm, t = 100 μs, N = 10. It is a very promising result, especially in light of multiple potential applications of compounds such as baicalein and wogonin. Glucuronide content was also slightly increased under the following treatment conditions: E = 1.75 kV/cm, t = 100 μs, N = 10; however, it was not as spectacular and less statistically relevant. Adjusting the parameters of electroporation is vital for achieving desired increases in concentrations of active compounds. However, these need to be further empirically adjusted until optimal parameters for both maximized flavonoid synthesis and plant growth are found. The potential blossom-promoting effect of electroporation is highlighted but further research is necessary to confirm it and elucidate the associated mechanisms.

## Data availability statement

The original contributions presented in the study are included in the article/**Supplementary Material**. Further inquiries can be directed to the corresponding authors.

## Author contributions

KG contributed to the conception and design of the study protocol, provided laboratory analysis, helped with statistical analyses, interpreted the data, and wrote the first version of the manuscript. AM interpreted the data, helped with results interpretation, and reviewed and corrected the final manuscript. SŚ contributed to the conception and design of the study protocol, provided laboratory analysis, helped with statistical analyses, interpreted the data, and finally reviewed the manuscript. All authors contributed to all manuscript versions, and read and approved the submitted version.
