# Supplementary material for: Electrostimulation improves plant growth and modulates the flavonoid profile in aeroponic culture of Scutellaria baicalensis Georgi
Source: Front Plant Sci. 2023 Mar 1;14:1142624. doi: 10.3389/fpls.2023.1142624 (PMC10014570; doi:10.3389/fpls.2023.1142624)
Supplement: Supplementary file 1 [file DataSheet_1.pdf]

Supplementary Material

Electrostimulation improves plant growth and modulates the flavonoid profile in aeroponic culture of *Scutellaria baicalensis*

Kajetan Grzelka[1], Adam Matkowski*[1,2] and Sylwester Ślusarczyk*[1]

*** Correspondence:**

Sylwester Ślusarczyk: sylwester.slusarczyk@umw.edu.pl

Adam Matkowski: pharmaceutical.biology@wp.eu

1 Supplementary Figures and Tables

1.1 Supplementary Figures

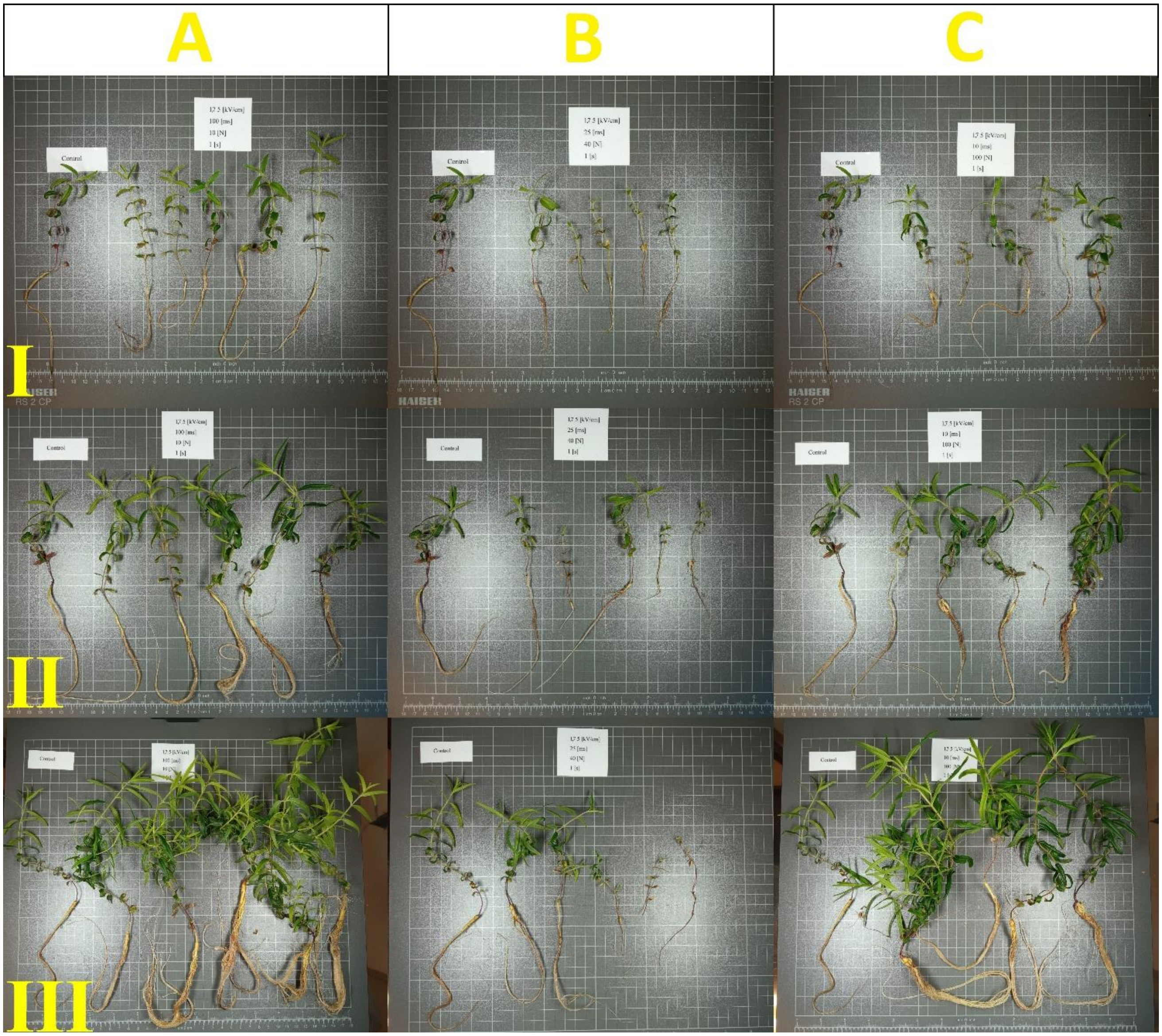

Supplementary Figure 1. Growth and development of *S. baicalensis* from Group 2 (E = 1.75 kV/cm) in 2-week intervals compared to a single specimen from the Control group shown on a 1cm x 1cm grid (row I – treatment day, row II – 2 weeks later, row III – 4 weeks later). Columns represent following subgroups: t = 100 µs, N = 10 (**A**), t = 25 µs, N = 40 (**B**), t = 10 µs, N = 100 (**C**)

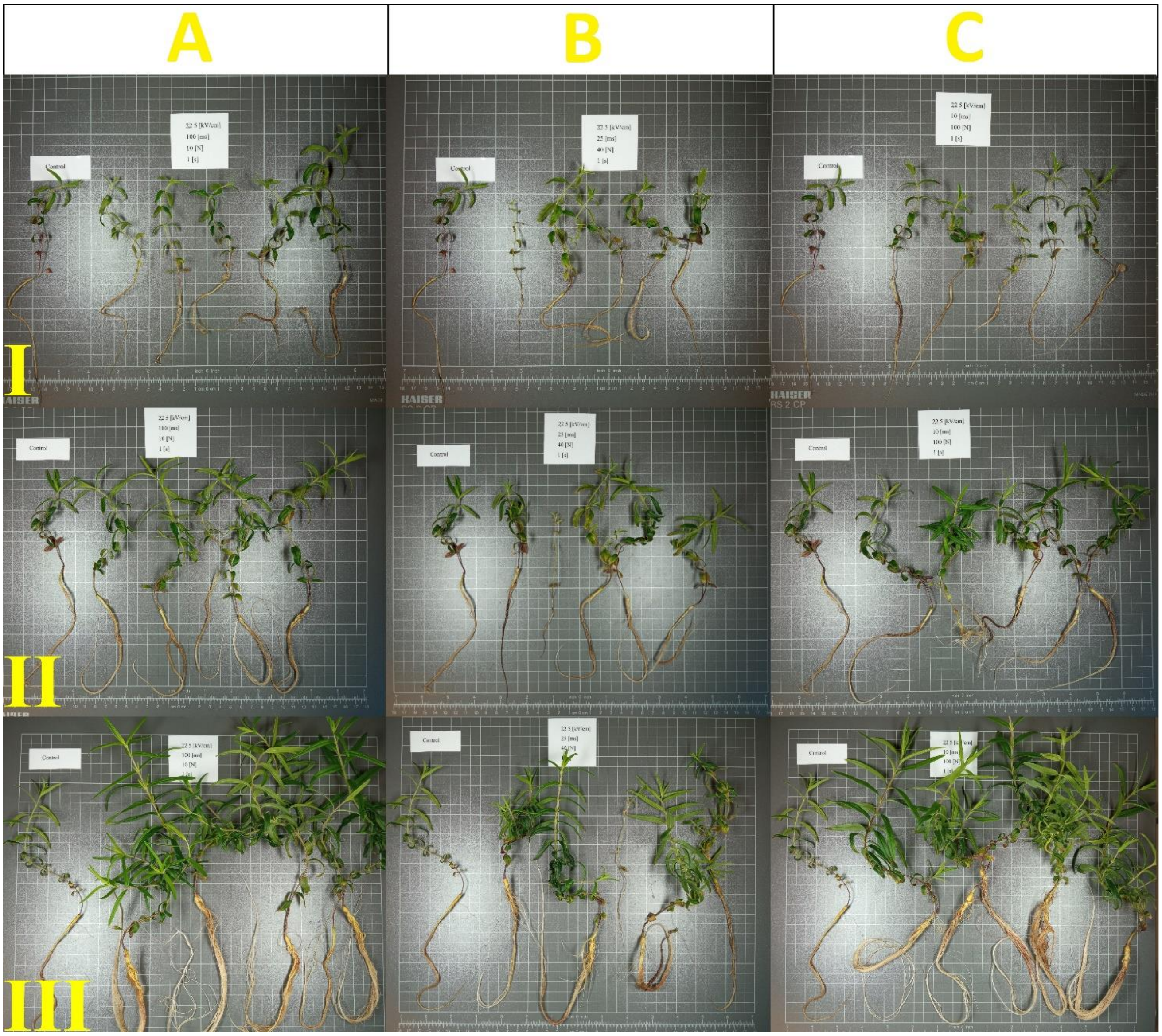

Supplementary Figure 2. Growth and development of *S. baicalensis* from Group 3 (E = 2.25 kV/cm) in 2-week intervals compared to a single specimen from the Control group shown on a 1cm x 1cm grid (row I – treatment day, row II – 2 weeks later, row III – 4 weeks later). Columns represent following subgroups: t = 100 μs, N = 10 (**A**), t = 25 μs, N = 40 (**B**), t = 10 μs, N = 100 (**C**)

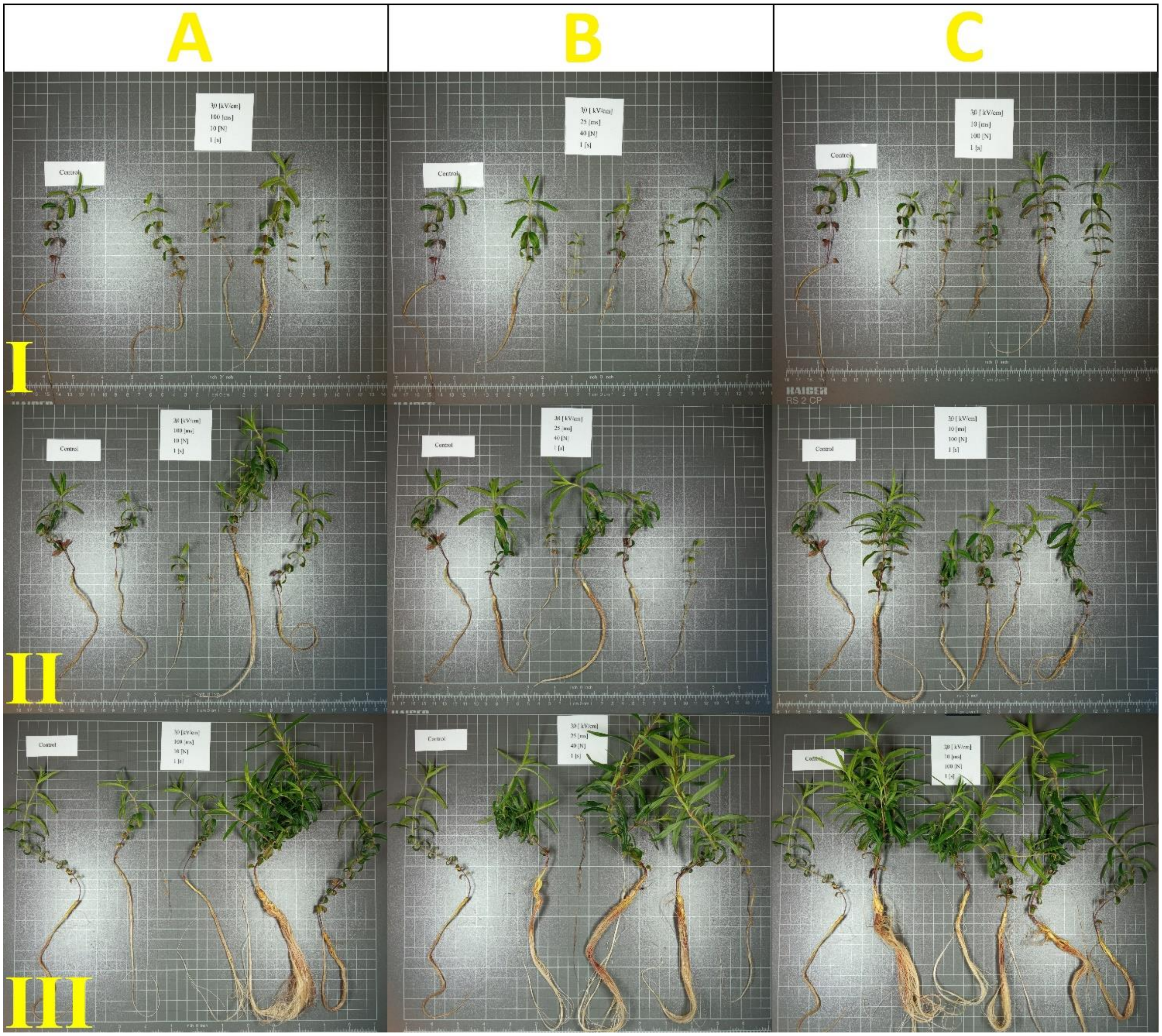

Supplementary Figure 3. Growth and development of *S. baicalensis* from Group 4 (E = 3.0 kV/cm) in 2-week intervals compared to a single specimen from the Control group shown on a 1cm x 1cm grid (row I – treatment day, row II – 2 weeks later, row III – 4 weeks later). Columns represent following subgroups: t = 100 μs, N = 10 (**A**), t = 25 μs, N = 40 (**B**), t = 10 μs, N = 100 (**C**)

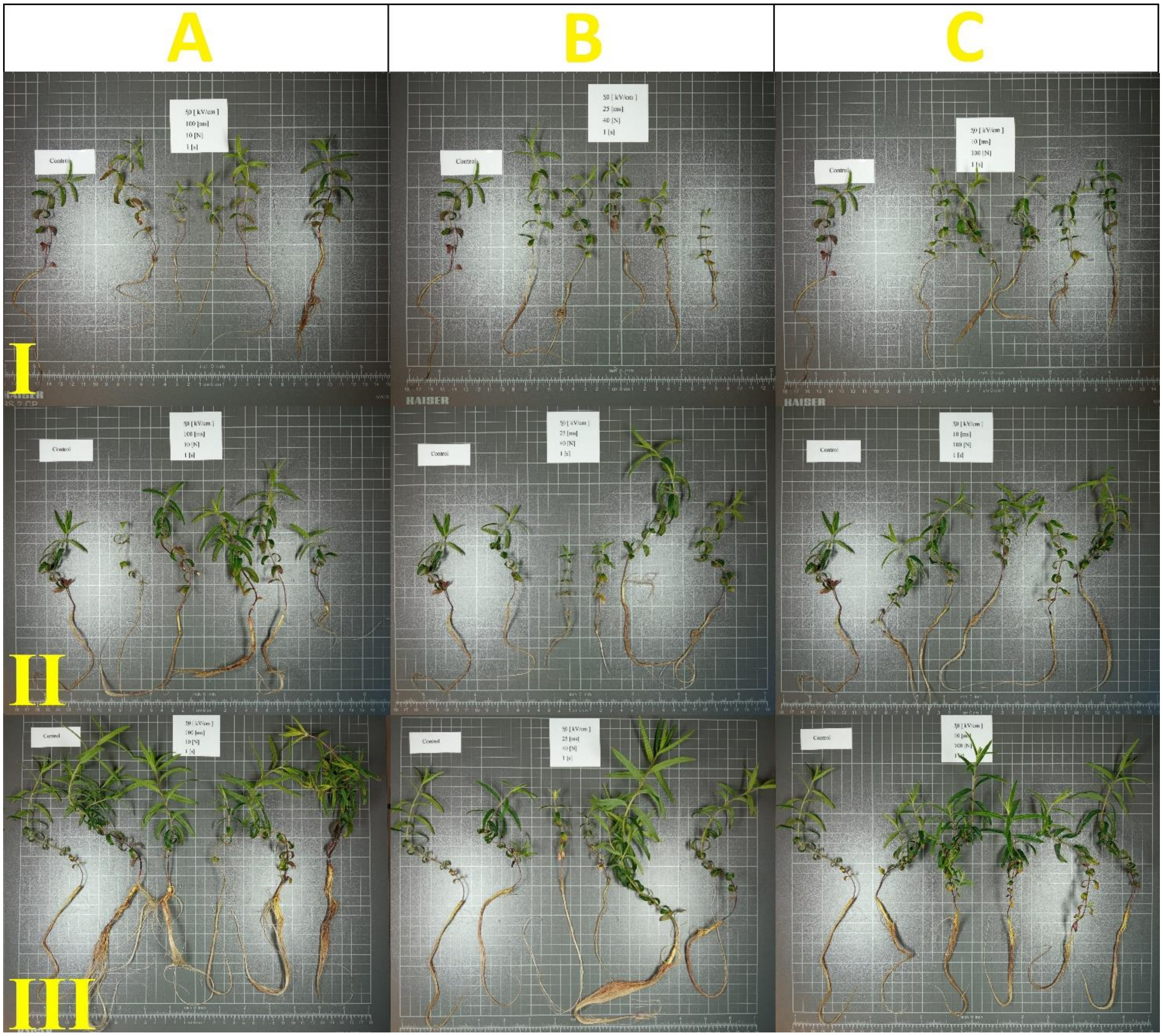

Supplementary Figure 4. Growth and development of *S. baicalensis* from Group 5 (E = 5.0 kV/cm) in 2-week intervals compared to a single specimen from the Control group shown on a 1cm x 1cm grid (row I – treatment day, row II – 2 weeks later, row III – 4 weeks later). Columns represent following subgroups: t = 100 μs, N = 10 (**A**), t = 25 μs, N = 40 (**B**), t = 10 μs, N = 100 (**C**)
